# Metabolic Reprogramming in Tumor Endothelial Cells

**DOI:** 10.3390/ijms231911052

**Published:** 2022-09-21

**Authors:** Melissa García-Caballero, Liliana Sokol, Anne Cuypers, Peter Carmeliet

**Affiliations:** 1 Laboratory of Angiogenesis & Vascular Metabolism, Center for Cancer Biology, VIB, B-3000 Leuven, Belgium; 2 Laboratory of Angiogenesis & Vascular Metabolism, Department of Oncology, KU Leuven, B-3000 Leuven, Belgium; 3 Laboratory of Angiogenesis & Vascular Heterogeneity, Department of Biomedicine, University Aarhus, 8000 Aarhus, Denmark; 4 Center for Biotechnology, Khalifa University of Science and Technology, Abu Dhabi 127788, United Arab Emirates

**Keywords:** tumor microenvironment, tumor angiogenesis, tumor endothelial cell metabolism, metabolic reprogramming

## Abstract

The dynamic crosstalk between the different components of the tumor microenvironment is critical to determine cancer progression, metastatic dissemination, tumor immunity, and therapeutic responses. Angiogenesis is critical for tumor growth, and abnormal blood vessels contribute to hypoxia and acidosis in the tumor microenvironment. In this hostile environment, cancer and stromal cells have the ability to alter their metabolism in order to support the high energetic demands and favor rapid tumor proliferation. Recent advances have shown that tumor endothelial cell metabolism is reprogrammed, and that targeting endothelial metabolic pathways impacts developmental and pathological vessel sprouting. Therefore, the use of metabolic antiangiogenic therapies to normalize the blood vasculature, in combination with immunotherapies, offers a clinical niche to treat cancer.

## 1. Introduction

The dynamic crosstalk between the different components of the tumor microenvironment (TME), composed of cancer cells, stromal cells, and extracellular matrix (ECM), is essential to promote cancer cell heterogeneity, clonal evolution, and multidrug-resistance mechanisms, leading to tumor progression and metastasis [1]. Among the stromal cells, endothelial cells (ECs) play a crucial role in the TME, and since the early 1970s, it was clear that tumors need to induce the vascular supply if they want to grow beyond a minimal size [2]. Thus, the activation of the angiogenic switch favors the vascular supply, whereby tumor blood vessels become functionally and morphologically heterogeneous and different from those of the normal vasculature [3,4]. Whereas the normal vasculature displays a hierarchal organization and the blood flows continuously, the tumor vasculature is not fully functional, chaotically distributed, and highly leaky, and blood often follows different paths through the same vessel [5]. Although less studied, tumor-associated lymphatic vessels also play an important role in the TME and in the establishment of distant metastases [6].

In the last few decades, it has been shown how cells in the TME have the ability to alter their metabolism in order to support the high energetic demands and favor rapid cancer cell proliferation [7,8,9,10]. Although the metabolic adaptations occurring in cancer cells have been intensively investigated, less focus has been given to the metabolic features of the other TME components. Interestingly, recent advances in the angiogenesis field have unraveled the plasticity and reprogramming of tumor EC metabolism, characterized by increased glucose uptake, glycolysis, diversion of glycolytic intermediates to the pentose phosphate, and alterations in the fatty acid (FA) and serine biosynthesis pathways [11,12,13,14]. Moreover, tumor vessel normalization, referring to the reversal of tortuous, leaky, and immature tumor vessels to more stable, functional and mature blood vessels, has emerged as a new therapeutic strategy to treat cancer [4,15,16]. Several studies have illustrated the benefits of antiangiogenic therapies that transiently remodel the abnormal vasculature in comparison with those that prune blood vessels [17,18]. Antiangiogenic therapies inducing vessel normalization restore tumor perfusion and enhance the efficacy of radiochemotherapy [4].

Immunotherapy has become an established treatment modality for cancer and has provided durable benefit in a subset of patients [19,20]. Therefore, the success of immune checkpoint blockade and adoptive cellular therapy, together with the evaluation of tumor EC metabolism to improve vessel normalization, will allow the development of combinatory approaches of antiangiogenic and immune modulators [17,21]. This review highlights the characteristics of the TME, tumor EC metabolic reprogramming, and the therapeutic possibilities of targeting tumor blood vessels.

## 2. Tumor Angiogenesis: A Pivotal Driver of Cancer Progression

Although solid tumor initiation does not rely on the formation of new blood vessels from the existing vascular bed (named angiogenesis), once the tumor is bigger than a few millimeters, it requires the formation of new blood vessels to ensure the supply of oxygen and nutrients, as well as to evacuate metabolic waste and carbon dioxide [2]. For several decades, sustained angiogenesis has been considered one of the hallmarks of cancer, as an excessive amount of proangiogenic stimuli in the TME maintains the “angiogenic switch” [22]. However, sprouting angiogenesis (SA) within tumors often results in nonproductive angiogenesis with abnormal blood vessels in structure and function [23]. In contrast to physiological conditions, in which the vasculature of the tissue efficiently supports blood distribution and transport from arteries to arterioles, and thereafter to capillaries, postcapillary venules, and veins, in tumors, there is a chaotic and nonhierarchical vasculature [4]. It is composed of abnormally dilated, tortuous, hyperpermeable, and hypoperfused vessels that lack proper perivascular coverage and tight EC junctions [24]. Thus, the presence of not fully functional vessels stimulates even more the production of proangiogenic factors (i.e., vascular endothelial growth factor (VEGF), fibroblast growth factor (FGF), placental growth factor (PlGF), angiopoietins, among others), consequently triggering a continuous self-reinforcing loop of nonproductive angiogenesis (reviewed by Cantelmo et al. [4] and Eelen et al. [23]).

The growing tumor creates high interstitial fluid pressure that collapses the blood vessels and induces fluid extravasation from the leaky tumor vessels [25]. This defective vasculature and the dysfunctional drainage, together with the hypoxia, low intratumoral pH and nutrient deprivation typical of the hostile TME, increase tumor aggressiveness and favor the escape of cancer cells [26,27]. Therefore, the tumor vasculature is not only required for the supply of nutrients and oxygen but also provides conduits for the dissemination of cancer cells from primary sites to other organs, giving rise to distant metastases. In addition, tumor vessels create a niche for cancer stem cells [28,29]. Besides cancer cells, cytokines and other factors in the TME, such as VEGF, can intravasate into the circulation and favor extravasation of metastatic cancer cells out of the blood vessels [30]. Immune cell infiltration in tumors is also determined by complex molecular and cellular mechanisms that limit or favor their penetration into the TME [31].

**Figure 1:**
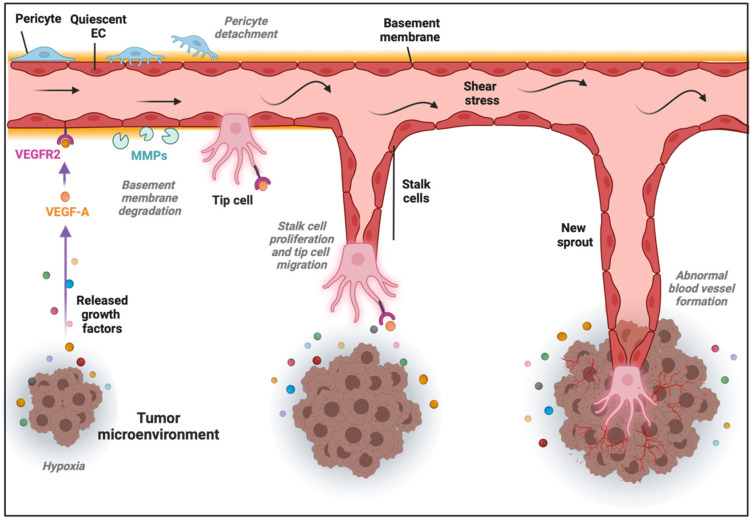
Sprouting angiogenesis in the tumor microenvironment (TME). Angiogenic stimuli such as hypoxia and proangiogenic growth factor gradients (in part produced by cancer cells) induce tip and stalk cell formation in a preexisting blood vessel. VEGF binds and activates its receptor VEGFR2, ECs become activated, and the detachment of pericytes and degradation of basement membrane and extracellular matrix (ECM) by matrix metalloproteinases (MMPs) take place. The tip cell becomes motile and starts to form lamellipodia and filopodia to migrate, while stalk cells proliferate to elongate the nascent vessel sprout in the TME.

The formation of new blood vessels within the TME can be accomplished through different mechanisms [32]. The most common is SA, in which blood vessels grow toward a gradient of proangiogenic factors, such as VEGF-A [33]. This stimulus induces the transition of ECs from a quiescent to an activated state through the acquisition of a more migratory and invasive phenotype. Consequently, the release of metalloproteinases (MMPs) triggers the detachment of pericytes and the degradation of the basement membrane and ECM, allowing ECs to proliferate, migrate, and initiate a new sprout. ECs present in the newly formed vessel experience dynamic phenotypic adaptations to form tip, stalk, and phalanx cells. Tip cells are polarized cells extending lamellipodia and filopodia specialized in sensing their environment and guiding the new sprout, while stalk cells are more proliferative cells elongating the nascent vessel (Figure 1) [34]. Once the tip cells belonging to different sprouts meet, they anastomose, develop lumens that are surrounded by a basement membrane and form a perfused vessel, after which ECs differentiate into quiescent phalanx cells. Phalanx cells establish a barrier to ensure blood perfusion and maintain redox homeostasis. In parallel, mesenchymal cells differentiate into smooth muscle cells and pericytes that participate in the subsequent stabilization of the new blood vessel [35]. Of note, recent findings from the analysis of ECs at the single-cell level demonstrated a higher degree of phenotypic complexity and heterogeneity, including activated postcapillary venules, immature (characterized by the lack of specific marker gene expression, but expressing activation markers and upregulating ribosomal gene expression consistent with an activated intermediate phenotype), transitioning (refers to an intermediate immature EC to tip EC), breach cells (those ECs that create a breach in the basal lamina, presumably involved in the initiation of vessel sprouting by tip cells), and neophalanx (characterized by the expression of markers of mature capillaries and arteries and the upregulation of a Notch signaling gene signature) EC phenotypes, all of them associated with vessel formation under pathological situations [36,37].

Besides SA, there is an alternative mechanism of tumor angiogenesis, named intussusceptive angiogenesis (IA) [38]. IA is characterized by the longitudinal splitting of one existing vessel to originate new ones in order to expand the capillary plexus. During the IA process, two opposite ECs make kissing contact to form a transluminal bridge and interendothelial junctions are reorganized to finally form an interstitial pillar core. The latter is finally invaded by pericytes, myofibroblasts and mesenchymal cells, splitting the original vessel into two independent ones [38]. From a metabolic point of view, IA might be less demanding than SA, considering the low EC proliferation and migration, together with the faster pace with which ECM and perivascular cells promote rapid generation of new vessels. Although the exact molecular mechanisms of IA remain largely unknown, Notch signaling inhibition in the existing vascular bed promotes pericyte detachment and extravasation of mononuclear cells, leading to rapid vascular expansion by IA, while disruption of Notch signaling in the leading edges of the nascent vessel triggers SA [39]. Other possible mechanisms of IA have been reviewed elsewhere [40,41,42].

Additional nonangiogenic mechanisms of tumor vascularization can take place [43]. One of them is vessel co-option, in which cancer cells hijack preexisting quiescent vessels from the surrounding parenchymal tissue to be incorporated into the tumor mass [44,45]. ECs in co-opted vessels proliferate less than in angiogenic ones and show low expression levels of the typical angiogenic markers [44]. Vessel co-option, associated with a poor prognosis in patients, has been observed in primary and metastatic tumors, especially in lung, brain, and liver tumors, and is a resistance mechanism to antiangiogenic therapies [46]. Notably, single-cell RNA sequencing of > 30,000 cells from a lung-vessel co-option tumor model revealed that co-opted tumor ECs (TECs) and pericytes display a similar transcriptome signature to their normal counterparts [45]. Moreover, matrix-remodeling macrophages might help cancer cells to co-opt vessels, and an M1-like macrophage subtype might be involved in the maintenance of quiescent ECs (QECs) [45]. In another nonangiogenic process, cancer cells can behave as ECs and generate a vessel-like meshwork, a mechanism named vascular mimicry [47]. Although first observed in human uveal melanoma, vascular mimicry appears in different aggressive metastatic cancers, such as prostate, breast, and lung cancers, glioblastoma, and melanomas [48,49,50]. It has been postulated that hypoxia can induce this mechanism via epithelial-to-mesenchymal transition, thus allowing the generation of channels lined by cancer cells and embedded in a rich ECM that ensure the tumor blood supply and their connection with the surrounding vascular network [51].

## 3. Metabolic Features of Normal and Tumor ECs

**Figure 2:**
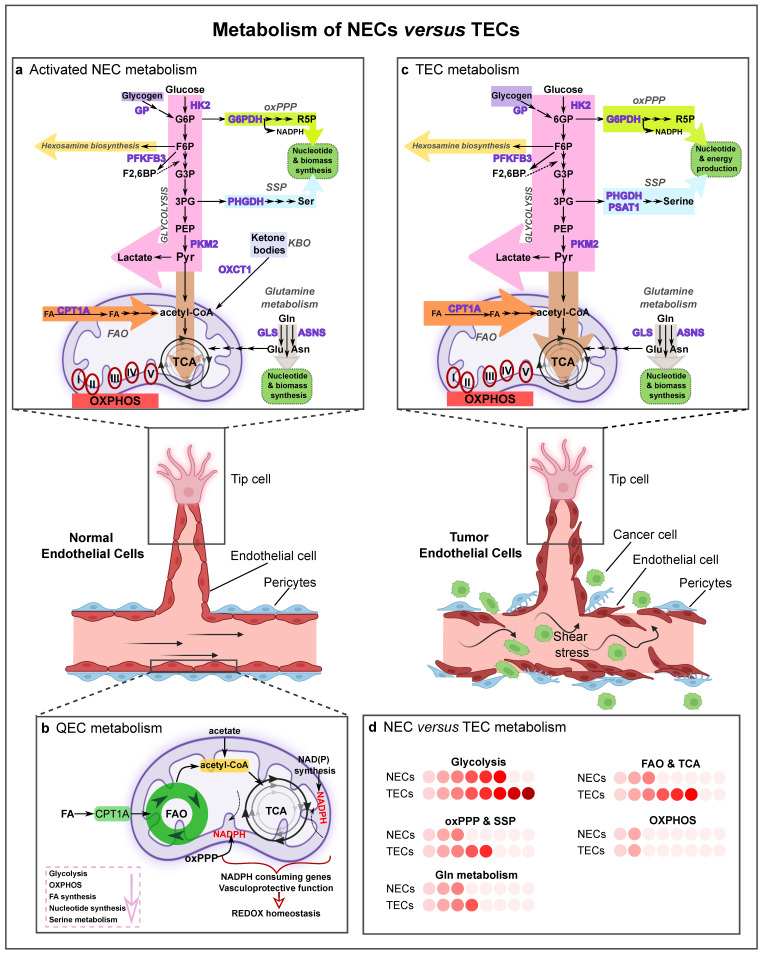
Metabolism of normal and tumor ECs. Graphical visualization of a normal and a tumor blood vessel with their metabolism. Normal vessels are characterized by a tightly adherent monolayer of ECs, intact basement membrane and rich pericyte coverage. Tumor vessels are lined by disorganized and structurally abnormal ECs and have a disturbed basement membrane with poor pericyte coverage, which leads to perturbed blood flow (shear stress) and allows cancer cells to enter the bloodstream. (**a**) Schematic representation of the main metabolic pathways in activated normal ECs (NECs). NECs are characterized by a high glycolytic flux, and use the oxidative pentose phosphate pathway (oxPPP), serine synthesis pathway and glutamine metabolism for nucleotide and biomass production. Acetyl-CoA derived from fatty acid oxidation (FAO) contributes to sustaining the TCA cycle (in conjunction with anaplerotic substrates) for deoxynucleotide (dNTP) synthesis during stalk cell proliferation. NECs retain functional mitochondria and OXPHOS, though minimally for ATP synthesis; instead, mitochondrial complex III is essential for EC proliferation and is critical for the maintenance of the NAD^+^/NADH ratio. In lymphatic ECs (LECs), the ketone-body oxidation pathway (KBO) generates acetyl-CoA which enters the tricarboxylic acid (TCA) cycle. (**b**) Quiescent ECs (QECs) display a lower rate of glycolysis, OXPHOS, fatty acid and nucleotide synthesis, and serine metabolism. They however increase FAO and oxPPP levels to regenerate NAPDH in order to maintain redox homeostasis and vascular barrier integrity. (**c**) Tumor ECs (TECs) are hyperglycolytic and have increased activity of the oxPPP, serine synthesis pathway, glutamine metabolism and FAO to sustain TEC proliferation. TECs can use glycogen as an alternative energy source and survive in nutrient-deprivation environments. In the figure, the arrow thickness represents the activity of the different metabolic pathways. (**d**) Activity of the selected pathways in activated NECs compared to TECs. Color code: the deeper the red, the higher the activity level of the metabolic pathway. Abbreviations: NEC, normal endothelial cell; GP, glycogen phosphorylase; HK2, hexokinase-2; G6P, glucose-6-phosphate; F6P, fructose 6-phosphate; F2,6BP, fructose 2,6-bisphosphate; PFKFB3, phosphofructokinase-2/fructose-2,6-bisphosphatase 3; G3P, glycerate 3-phosphate; 3PG, 3-phosphoglycerate; PEP, phosphoenolpyruvate; PKM2, pyruvate kinase M2; Pyr, pyruvate; G6PDH, glucose-6-phosphate dehydrogenase; R5P, ribose-5-phosphate; oxPPP, oxidative pentose phosphate pathway; PHGDH, phosphoglycerate dehydrogenase; Ser, serine; SSP, serine synthesis pathway; PSAT1, phosphoserine aminotransferase; KBO, ketone-body oxidation pathway; OXCT1, 3-oxoacid CoA-transferase 1; Gln, glutamine; GLS, glutaminase; Glu, glutamate; Asn, asparagine; ASNS, asparagine synthetase; FA, fatty acid; CPT1A, carnitine palmitoyltransferase 1A; FAO, fatty acid oxidation; TCA, tricarboxylic acid cycle; OXPHOS, oxidative phosphorylation; QEC, quiescent endothelial cell; TEC, tumor endothelial cell.

ECs can form new vessels in physiological conditions, for example, during development or as a response to a transitory injury, and remain quiescent in adults [52,53]. However, ECs can become rapidly activated during pathological angiogenesis [52,53]. The EC phenotype is not in a preset or fixed state, but can dynamically switch between phenotype states in response to growth factors, with VEGF as the main regulator [54]. In SA, the growth factor-induced angiogenic switch from a quiescent phalanx phenotype to an angiogenic state is codetermined by a metabolic switch [23]. The three main subtypes mentioned previously (tip, stalk, and phalanx cells) differ in their needs regarding energy and biomass production [55,56]. While QECs mainly use their metabolism to maintain redox homeostasis, angiogenic ECs (tip and stalk ECs) particularly need efficient ways of ATP and biomass production (Figure 2a,b) [23]. Thus, ECs differ in their metabolic features, and recent studies have reported that EC metabolism is disturbed and changed in diseased conditions, such as cancer, making the TEC metabolism a potential target to normalize those perturbations. The next sections describe key findings on EC metabolism focusing on sprouting angiogenesis, on which more information is available.

### 3.1. Normal EC (NEC) Metabolism

#### 3.1.1. Quiescent ECs

In healthy adults, ECs remain quiescent and are responsible for tissue perfusion, prevent thrombosis and vascular inflammation, preserve vasoregulation and control barrier function [10,57]. In their environment, QECs are exposed to high oxygen levels and as a consequence to high oxidative stress, which is known to cause several EC dysfunctions [57,58]. The metabolism of QECs is still not thoroughly investigated. However, recent studies show that QECs are not hypometabolic and that they upregulate fatty acid oxidation (FAO) about threefold higher than proliferating ECs, but with different aims [59]. Instead of using FAO to sustain the tricarboxylic acid (TCA) cycle (in conjunction with other anaplerotic metabolites) to produce biomass [56], they rather keep the TCA cycle active for redox homeostasis through regeneration of NADPH and vasculoprotection against oxidative stress [59] (Figure 2b). Importantly, loss of carnitine palmitoyl transferase 1a (CPT1a—a rate-controlling enzyme of FAO) results in EC dysfunction in vivo and promotes LPS-induced vascular inflammation and inflammatory bowel disease in mice [59]. FAO is regulated by Notch signaling, which reprograms FAO in QECs. In ECs, Notch also inhibits DNA synthesis by inducing the cell cycle inhibitor p27KIP1 and suppression of nuclear translocation of cyclinD1-CDK4, drivers of cell cycle progression [60,61]. Thereby, the cell-cycle inhibitory signaling prevents QECs from anabolic nucleotide synthesis [59]. An opposite metabolic activity (downregulation) has been observed in QECs for several other pathways, such as glycolysis, TCA cycle, serine biosynthesis, oxidative phosphorylation (OXPHOS), and nucleotide and FA synthesis (Figure 2b) [59].

It has been reported that FOXO1 acts as a gatekeeper of QECs and is necessary for vascular homeostasis [62]. It belongs to a subgroup of the FOX transcription factor family and is a downstream mediator of Akt [62]. FOXO1 has been identified as an important metabolic controller and negative regulator of EC proliferation, and maintains EC quiescence by inhibition of the key transcription factor MYC, glycolysis and mitochondrial function [63,64]. Recently, it has been documented that PKM2 in QECs, besides its canonical pyruvate kinase activity, maintains the vascular barrier function and modulates an inflammatory pathway dependent on NF-kB signaling [65].

#### 3.1.2. Activated ECs

GLYCOLYSIS: When QECs are activated, they become addicted to glucose, but only a small part of pyruvate generated by glycolytic activity enters oxidative metabolism. Around 85% of the intracellular ATP is produced by converting glucose to lactate [52,55]. There are several reasons for their addiction to glycolysis: (i) to generate ATP faster than via OXPHOS, since tip ECs need to rapidly produce ATP. Local glycolysis-derived ATP production at filopodial and lamellipodial protrusions controls migratory speed and directionality [34,55]; (ii) to preserve oxygen for other perivascular cells [66,67]; (iii) to allow ECs to form new blood vessels in hypoxic regions [55]; and (iv) to lower the production of reactive oxygen species (ROS) (ECs are exposed to high oxygen levels) [55]. EC glycolysis is stimulated by 6-phosphofructo-2-kinase/fructose-2,6-bisphosphatase 3 (PFKFB3), as the conversion of fructose-6-phosphate (F6P) to fructose-1,6-bisphosphate (F1,6P_2_) by 6-phosphofructo-1-kinase (PFK-1), a rate-limiting step of glycolysis, is enhanced by the allosteric activator fructose-2,6-bisphosphate (F2,6P_2_), the product of PFKFB3 [55]. A second glycolytic regulator (and limiting enzyme) in ECs is hexokinase 2 (HK2), which phosphorylates glucose to glucose-6-phosphate (Figure 2a) [68]. Together, these glycolytic regulators are repressed by KLF2, a transcription factor that becomes activated upon laminar shear stress [69]. Thus, the phalanx cell state is sustained by the blood flow in a healthy condition, partially due to the inhibition of the glycolytic metabolism (via KLF2 and by FOXO1 inhibiting MYC), while the glycolytic flux is increased when ECs switch to an angiogenic EC phenotype [55]. VEGF stimulation controls this switch by rewiring EC metabolism to meet the energetic needs of the activated angiogenic state. PFKFB3 is upregulated in a VEGF-dependent manner and activates PFK-1, which is paralleled by an increase in the glycolytic flux [70]. More particularly, VEGF stimulation of ECs results in elevated glycolysis, by increasing the expression levels of the glucose transporter 1 (GLUT1/SLC2A1) (via activating phosphoinositide 3-kinase (PI3K)-Akt signaling) and of glycolytic enzymes, such as lactate dehydrogenase-A (LDH-A) and PFKFB3. Interestingly, PFKFB3-driven glycolysis can be reduced by activation of Notch receptor signaling, which is a pro-stalk phenotype regulator. This suggests that high glycolytic activity is a feature particularly characteristic of the tip cells [55]. In accordance, genetic loss or pharmacological inhibition of PFKFB3 diminishes the tip cell behavior, and differentiation to the stalk to tip cell phenotype can be promoted by overexpression of PFKFB3 in in vitro and in vivo sprouting experiments, even in conditions of *Notch* overexpression, indicating that glycolysis can overcome genetic instructions modulating EC specification [55,70]. Additionally, PKM2 maintains EC proliferation via suppression of NF-kB/p53 signaling [65,71]. These seminal studies were the first to demonstrate that EC metabolism is not merely a secondary response to growth factor stimuli, but that a change in EC metabolism (even without a change in angiogenic stimuli) suffices to alter EC differentiation and vessel sprouting.

OTHER GLUCOSE PATHWAYS: Activated ECs use other metabolic ways to process glucose as well (Figure 2a). When ECs are deprived of external glucose, they can also use internal glucose, stored as glycogen, which is converted by glycogen phosphorylase to glucose-1-phosphate, and then by phosphoglucomutase to glucose-6-phosphate (G6P) to enter glycolysis [72,73,74]. Alternatively, G6P can also be used in a branch of the pentose phosphate pathway (PPP), the oxidative PPP (oxPPP), to generate ribulose-5-phosphate (Ru5P) and NADPH. Ru5P, as a precursor of ribose-5-phosphate (R5P), is important for nucleotide biosynthesis, while NADPH is crucial to regenerate reduced glutathione (GSH) from its oxidized form (GSSG) for redox homeostasis [75]. The hexosamine biosynthetic pathway (HBP), another side branch of glycolysis generating uridine diphosphate-N-acetylglucosamine (UDP-GlcNAc), a key substrate for protein glycosylation, has been proposed as a nutrient sensor for angiogenesis modulation, since the glycosylation status of, for instance, VEGFR1 and Notch1, codetermines their function (Figure 2a) [76,77].

OXPHOS: Despite the importance of glycolysis for NECs, several studies have reported that mitochondrial complex III is essential for EC proliferation and is critical for the maintenance of the NAD^+^/NADH ratio. This denotes that mitochondrial respiration has an important role in NEC metabolism [78,79], even though ECs rely minimally on mitochondrial OXPHOS for ATP generation (an estimated 15% derives from OXPHOS) [55], and mitochondria serve as biosynthetic hub rather than a powerhouse organelle in ECs [67,80]. Still, mitochondria in ECs are not dysfunctional and remain fully coupled to ATP synthesis, implying normal flow through the electron transport chain.

FAO and TCA CYCLE: FA metabolism is also essential for EC proliferation [70,81]. In EC and lymphatic ECs (LECs), FAO regulates the TCA cycle through CPT1a, the transporter responsible for the FA import into mitochondria [82,83]. Once FAs enter β-oxidation, they are metabolized to acetyl-CoA, which helps to sustain the TCA cycle in conjunction with anaplerotic substrates, resulting in the production of the precursors glutamate and aspartate for deoxynucleotide (dNTP) synthesis during EC proliferation [56]. Furthermore, in LECs, FAO promotes the venous-to-lymphatic EC differentiation via epigenetic mechanisms [56,82]. During the developmental transdifferentiation of venous ECs (VECs) into LECs, the master transcription factor PROX1 upregulates CPT1a expression and thus FAO levels, resulting in elevated acetyl-CoA levels [56,82]. Acetyl-CoA is then used by histone acetyltransferase p300, and PROX1-p300 interaction favors histone acetylation at lymphangiogenic genes (i.e., *VEGFR3*, *PROX1*), promoting lymphangiogenesis [56,82].

FA SYNTHESIS: Also worth mentioning is the role of the endothelial FA synthase (FASN), an enzyme that catalyzes the production of palmitate, using malonyl-CoA as substrate. FASN, in addition to mediating de novo lipid synthesis, regulates angiogenesis via malonylation of key targets [12]. FASN promotes sprouting in vitro and its downregulation decreases EC proliferation without affecting EC migration. Inhibition of FASN increases the levels of malonyl-CoA without a relevant decrease in the cellular palmitate levels, likely because ECs can readily take up palmitate from the milieu. Elevation of malonyl-CoA increases protein malonylation, a posttranslational protein modification, including malonylation of mTOR, which decreases its activity and impairs EC growth [12].

AMINO ACID METABOLISM: EC proliferation and vascular expansion is also maintained by amino acid metabolism. Glutamine, the amino acid that ECs metabolize the most, contributes to citrate production by means of reductive carboxylation, to anaplerotically replenish carbons into the TCA cycle, and provides nitrogen for the biosynthesis of proteins and nucleotides [84,85]. Therefore, glutamine depletion or glutaminase 1 (GLS1) inhibition, not only impairs EC proliferation, migration and biomass synthesis but also promotes EC senescence, reduces mTOR enzymatic activity, and increases the expression of genes related to endoplasmic reticulum (ER) stress [70,84]. Furthermore, glutamine contributes to asparagine synthesis by providing nitrogen [84]. Asparagine can be used as substrate for protein synthesis. Interestingly, cellular levels of asparagine are low in proliferating cells, and asparagine amination relies on glutamine, suggesting that asparagine synthesis might play a role as a rheostat in sensing the availability of TCA cycle intermediates and the supply of reduced nitrogen [84,86]. Serine is another amino acid metabolized by ECs. ECs not only take up this amino acid but also synthetize it in a three-step enzymatic reaction, controlled by the key enzyme phosphoglycerate dehydrogenase (PHGDH). Notably, serine interconnects glutamine and glucose metabolism because it is synthesized from α-nitrogen of glutamate and from the glycolytic intermediate 3-phosphoglycerate [87]. Further, serine contributes to one-carbon metabolism for redox homeostasis and purine nucleotide synthesis [88]. EC-specific deletion of PHGDH was found to deplete the intracellular heme pool, causing reduced activity of the heme-containing oxidative phosphorylation complexes III and IV and subsequent defective mitochondrial respiration leading to vascular defects and neonatal lethality [14]. The defective mitochondrial respiration in PHGDH-defective ECs also compromised the activity of the mitochondria-associated enzyme dihydroorotate dehydrogenase (DHODH), which generates precursors for pyrimidine nucleotides, and requires a functional electron transport chain for its activity [14]. Loss of the PHGDH enzyme thus resulted in the overall reduction of nucleotides and of heme synthesis [14].

KETONE-BODY OXIDATION PATHWAY: Interestingly, another study has demonstrated that lymphangiogenesis relies on acetyl-CoA, derived from the ketone-body oxidation pathway [1,83]. Loss of 3-oxoacid-CoA-transferase-1 (OXCT1, the rate-limiting enzyme of the ketone-body oxidation pathway) in LECs reduced in vitro sprouting and impaired in vivo lymphangiogenesis, both in development and in diseased conditions, while the supplementation of ketone bodies caused the opposite effects [83].

### 3.2. Tumor EC (TEC) Metabolism

TECs have a higher glycolytic rate than NECs and rely on glycolysis as the main source of ATP production [52]. Compared with NECs, TECs are characterized by increased glucose uptake and diversion of glycolytic intermediates to the PPP and serine biosynthesis pathways for the synthesis of nucleotides (Figure 2c,d) [13]. Their hyperglycolytic phenotype is likely due to the combination of the modifications in the expression of glycolytic enzymes and the release of proangiogenic (especially VEGF) and proglycolytic factors, both induced by the harsh hypoxic conditions in the TME [13,27]. Upon hypoxia exposure and through HIF1α stabilization, TECs upregulate the expression of GLUT1 and the abovementioned glycolytic activator PFKFB3, regulating a switch from OXPHOS to glycolysis [13,41,89,90,91,92]. TECs also increase glycolysis via upregulation of VEGF by cyclooxygenase 2 (COX2) [11]. Additionally, lactate accumulation in hypoxic conditions can influence EC metabolism and cause lactate uptake by ECs. Lactate acts as a signaling messenger, stimulating angiogenesis via VEGF signaling through HIF1α and the PI3K/AKT pathway [93]. Furthermore, lactate can be further oxidized to pyruvate, which induces angiogenesis via ROS-mediated stimulation of NF-κB and interleukin (IL)-8 [94]. An inflammatory response and ER stress can also be induced in ECs due to lactate accumulation [95].

These findings may have therapeutic implications. Indeed, disturbing PFKFB3 function in TECs, both by endothelial-specific genetic loss or by general pharmacological inhibition with the small molecule 3-(3-pyridinyl)-1-(4-pyridinyl)-2-propen-1-one (3PO), arrested EC sprouting and proliferation [55,96], decreased EC leakiness, tightened the vascular barrier, and restored perfusion, inducing tumor vessel normalization [4,13]. Notably, a reduction in glycolytic flux by 15–20% in TECs using 3PO at low doses in tumor-bearing mice induced tumor vessel normalization, and secondarily reduced metastasis and improved chemotherapy responses, while 3PO treatment with a maximally tolerable dose reduced TEC glycolysis to the extent that it caused vessel disintegration, further favoring tumor cell extravasation and dissemination [13]. These results highlight that proper dosage of antiangiogenic drugs is critical, and targeting EC metabolism should aim at reestablishing the glycolytic flux in TECs to levels comparable to those observed in healthy NECs, rather than killing TECs, which finally favors the occurrence of undesired effects and toxicity and increases the risk of metastasis [97].

Besides glycolysis, TECs reprogram their metabolism also via alternative routes. For example, TECs retain functional mitochondria [98] and OXPHOS provides an added value due to the use of alternative substrates for energy and mass production in order to support TEC proliferation [99,100]. TGF-β1 and RAF/MEK/ERK-signaling pathways, implicated in cancer progression, also induce glutamine metabolism in TECs, suggesting a role in TECs [101,102]. However, different from observations in cancer cells [103], the EC proliferation defect in glutamine starving conditions cannot be rescued by supplementation with antioxidants or replenishment of the TCA cycle alone, indicating that ECs rely differently and perhaps also more prominently on glutamine, and additionally on asparagine compared to cancer cells [84]. TECs show alterations in FA and serine biosynthesis pathways, such as upregulation of FASN, PHGDH, and phosphoserine aminotransferase 1 (PSAT1) [1]. In addition, since FAs are used by proliferating ECs to sustain the TCA cycle for de novo nucleotide synthesis [34], such metabolic properties could have therapeutic implications, which remain to be studied in the setting of cancer.

TECs are involved in both nutrient replenishment and metastasis of the stressed and starving cancer cells. In the TME, TECs themselves are also subjected to harsh conditions, such as hypoxia, starvation, low glucose, and low blood flow. Autophagy is upregulated in TECs in response to the mentioned extracellular stress [104], and recently, the role of autophagy in the tumor vasculature is receiving more attention [90]. Autophagy is controlled by several regulators, including mTOR and AMP-activated protein kinase-α (AMPKα). Compared to NECs, TECs may adapt their autophagy/lysosomal activity to reduce the damaging effect of hypoxia. By activating autophagy, TECs can use glycogen as an important backup energy source and survive in nutrient-deprived environments [73]. As mentioned before, ECs can store glycogen in a high-glucose situation for its mobilization in case of starvation and stressful conditions. Indeed, under hypoxic conditions, around 90% of glycogen is converted to G6P, which then can be directly incorporated in glycolysis and used to maintain EC proliferation and migration [90].

Although further studies will be required to uncover the potential of targeting TEC metabolism for tumor vessel inhibition or normalization, there are already some results showing the effects of compounds interfering with EC metabolism. For example, in animal models of ocular diseases, such as retinopathy of prematurity, treatment with low-dose 3PO decreased retinal neovascularization [96]. Targeting FAO by inhibiting CPT1a with etomoxir also showed benefit in a model of retinal neovascularization and in a model of corneal injury-induced lymphangiogenesis [55,56,82].

## 4. Metabolism of ECs at the Single-Cell Level

**Figure 3:**
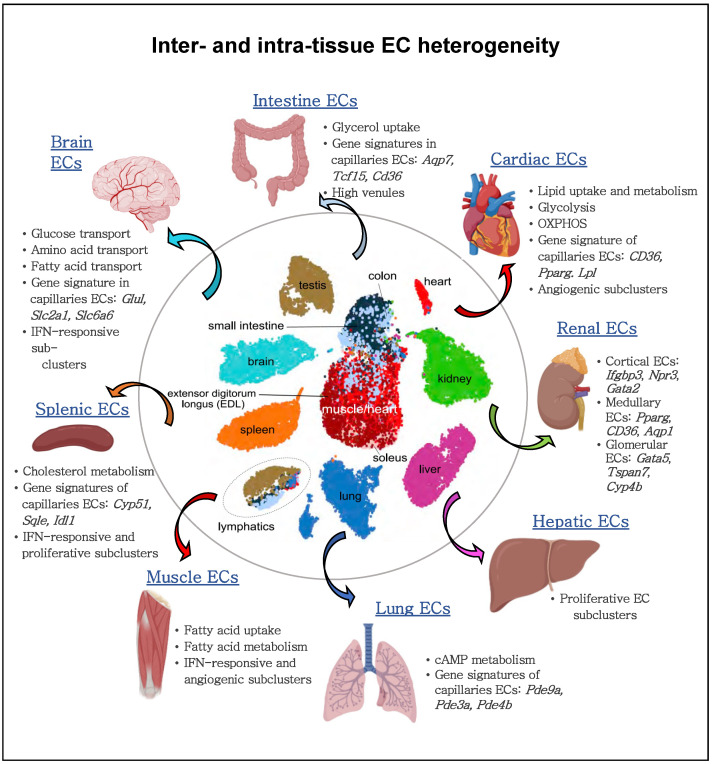
Inter- and intra-EC heterogeneity based on single-cell transcriptome profiles. Quiescent ECs isolated from different murine tissue types show heterogeneity between vascular beds and tissue-specific metabolic adaptations within the same organ. Quiescent cardiac and muscle ECs show an enriched gene signature implicated in lipid uptake and metabolism; lung ECs upregulate genes involved in cAMP metabolism; brain ECs upregulate the expression of glucose, amino acid and fatty acid transporters; splenic ECs increase the expression of cholesterol metabolism genes. Moreover, ECs from arteries, capillaries, and veins within the same organ display differential metabolic gene expression. For gene names, see the main text.

Recent studies highlighted EC heterogeneity at the single-cell level [105], including metabolic gene signature [34,36,37,105,106,107,108]. A murine single cell atlas from 11 healthy tissues unveiled the heterogeneity of ECs with specification per tissue type and identified heterogeneous metabolic gene patterns in ECs between tissues and between vascular beds (Figure 3) [105]. For instance, capillary ECs were found to upregulate genes involved in uptake and metabolism of glycerol and FAs. Also, an unbiased approach revealed that distinct metabolic genes were upregulated in ECs from different tissues and codetermined the tissue-grouping phenomenon of ECs. For example, spleen ECs upregulated cholesterol metabolism genes in line with the spleen’s suggested role in plasma cholesterol regulation, and cardiac and muscle ECs expressed higher levels of genes involved in FA uptake and metabolism (Figure 3) [105].

Although EC metabolism is an emerging target for antiangiogenic therapies in tumors and other angiogenesis-dependent diseases like choroidal neovascularization (CNV), still little is known about individual EC metabolic transcriptomes in normal and disease contexts. Rohlenova et al. compared single murine choroidal ECs upon induction of CNV (CNV-ECs) with single murine tumor lung ECs, and identified congruent marker gene expression across tissues and diseases for distinct EC phenotypes, thus indicating a similar angiogenic mechanism [37]. The study showed that the expression of genes involved in ATP synthesis, membrane transport and glycolysis is dynamically regulated during differentiation from quiescent vein EC to angiogenic EC phenotypes. The biggest changes in carbon metabolism gene expression were observed in the most angiogenic subtypes, suggesting that those ECs have the highest metabolic demands. Both in tumor and CNV, proliferating ECs upregulated the expression of genes involved in one-carbon metabolism, TCA cycle, OXPHOS, and nucleotide synthesis. In contrast, glycolytic gene expression was highly upregulated in all angiogenic TEC populations (proliferating, tip, and immature) and was elevated in proliferating CNV-ECs, but less upregulated in other angiogenic CNV-EC phenotypes (tip and immature) [37]. Thus, metabolic demands of proliferating ECs during the angiogenic switch seem to be independent of diseases and tissue, while the adaptations of the metabolic gene signature in other EC subtypes appear to be more plastic. The differences in metabolic gene expression between other EC phenotypes were more pronounced in TECs than in CNV-ECs, possibly due to the harsh, nutrient-scarce conditions in the TME. For example, capillary TECs upregulated genes controlling lipid uptake (possible role in the quiescence switch; see also section on NEC above) whereas venous TECs upregulated transcripts involved in prostaglandin metabolism, suggesting a role in vascular inflammation, sprouting or vasoregulation [37]. Finally, through an integrated approach, this study identified the metabolic targets SQLE and ALDH18A1 as proangiogenic targets.

Another study looked at single cells collected from patients with colorectal, lung, ovary or breast cancer and constructed a pan-cancer blueprint of stromal cell heterogeneity using different scRNA-seq and protein-based technologies in order to investigate the heterogeneity and similarities between cancers in different organs [106]. Similarly to the abovementioned studies, tip cells expressed a gene signature of heightened glycolysis and OXPHOS. On the other hand, the LEC metabolic signature suggested an increased FAO gene signature, which was previously described to be essential in lymphangiogenesis [41,106].

Another single-cell study compared NECs versus TECs isolated from cervical cancer and revealed increased expression of several metabolic genes in TECs [107]. NECs were characterized by upregulation of genes involved in basic biological functions related to the vascular system and EC development, EC migration and angiogenesis [107]. By contrast, TECs had gene signatures, enriched for peptidase activity, proteolysis regulation, extracellular structure organization, xenobiotic metabolism, PPAR signaling pathway, amino acid biosynthesis, carbon metabolism, and glycolysis/gluconeogenesis. Other enriched pathways in TECs included genes involved in DNA replication, TCA cycle, and cell cycle, crucial for cell proliferation. Interestingly, the expression of pathways involved in immune regulation were underexpressed in TECs, thus possibly contributing to tumor immune tolerance [107].

## 5. Modulation of Tumor Endothelial Cell Metabolism as an Innovative Therapeutic Approach

**Figure 4:**
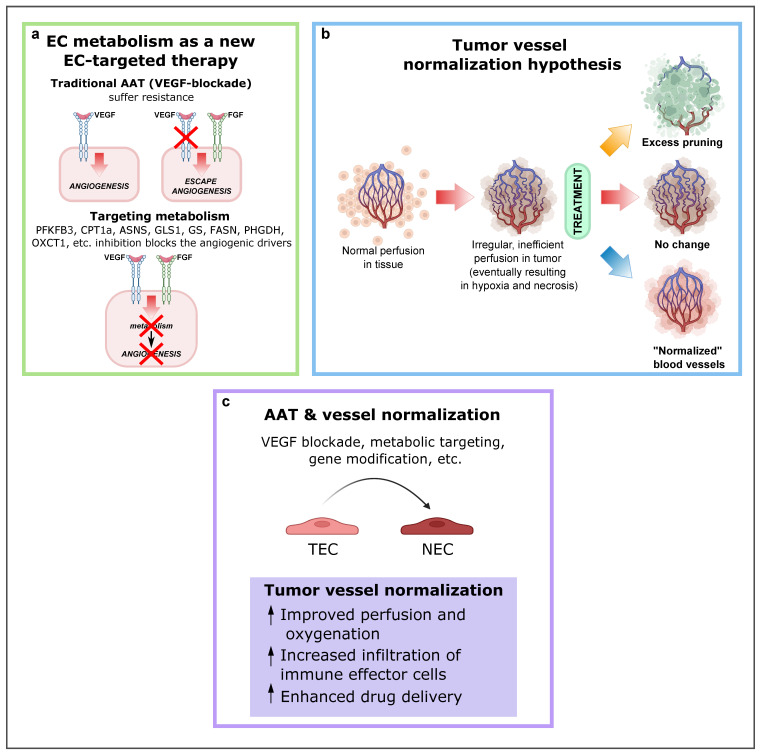
EC metabolism as a new EC-targeted therapy approach, tumor vessel normalization and alternative improved AAT in combination with other antitumor drugs. (**a**) Targeting EC metabolism as an alternative method to block pathological angiogenesis. While current anti-VEGF therapy compensatorily induces other angiogenic drivers (alternative growth factors), targeting EC metabolism, an engine driving angiogenesis, impairs vessel growth, regardless of how many angiogenic signals are present. Reproduced from [1] using BioRender.com. (**b**) Depending on the type and intensity of antiangiogenic therapy (AAT), the tumor vasculature can be pruned, leading to decreased blood perfusion (top); not respond to therapy (center); or become “normalized,” resulting in increased blood perfusion (bottom). Reproduced from Sorensen et al. [120] using BioRender.com. (**c**) Consequences and effects of AAT and vessel normalization. Abbreviations: EC, endothelial cell; VEGF, vascular endothelial growth factor; PFKFB3, phosphofructokinase-2/fructose-2,6-bisphosphatase 3; CPT1A, carnitine palmitoyltransferase 1A; ASNS, asparagine synthetase; GLS1, glutaminase; GS, glutamine synthase; FASN, fatty acid synthase; PHGDH, phosphoglycerate dehydrogenase; OXCT1, 3-oxoacid CoA-transferase 1; AAT, antiangiogenic therapies; TEC, tumor endothelial cell.

As previously described, a mechanism by which tumors can secure their blood supply is SA [53,109,110]. Years ago, this discovery led to the development of antiangiogenic treatments with the underlying idea that cutting off the blood supply would starve cancer cells to death. In some cancer types, this treatment works, but in most cases resistance eventually develops. Notably, VEGF or VEGF receptor blockade suffers from intrinsic refractoriness, acquired resistance, and inadequate efficacy [111]. Therefore, instead of targeting angiogenic signals, targeting EC metabolism to treat pathological angiogenesis has emerged as a new approach (Figure 4a), and several key metabolic enzymes, such as PFKFB3, CPT1a, asparagine synthetase (ASNS), GLS1, GS, FASN, PHGDH, OXCT1, and mitochondrial complex III enzymes, have been already investigated as potential targets to inhibit pathological (lymph)angiogenesis without causing systemic toxicity.

TECs are more glycolytic than NECs, and they upregulate PFKFB3 expression. Thus, lowering PFKFB3-driven glycolysis in TECs normalizes the abnormal tumor vasculature [63,67]. Both pharmacological and genetic inhibition can be used to block PFKFB3. Partial pharmacological inhibition with 3PO (targets PFKFB3) resulted in an in vivo and in vitro reduction of 35–40% of glycolysis in ECs [96]. Besides 3PO, other (more aggressive) glycolysis blockers have been tested, such as 2-deoxy-d-glucose (2DG), which reduced glycolysis up to 80%, but also caused cell death and an unhealthy morphology, while 3PO, on the other hand, did not provoke cell death. Since it has been demonstrated that only partial blockade of PFKFB3 is beneficial, drug dynamics should always be considered and not only the metabolic target itself.

Pharmacological blockade of CPT1a with etomoxir or perhexiline has shown promising antiangiogenic and antilymphangiogenic effects [56,82,112,113]. In the ocular retinopathy model (ROP model) in mouse pups and in the corneal model of injury-induced lymphangiogenesis, etomoxir reduced proliferating ECs and pathological vascular growth [56,82].

Several studies explored glutamine involvement in EC migration, sprouting and proliferation [63,84]. In particular, in vitro and in vivo GLS1 genetic deletion or GLS1-specific inhibitor (CB-839) inhibited EC migration, sprouting, and proliferation [84]. In the tumor environment, which is characterized by hypoxia, ER stress, and amino acid or glucose deficiency, ECs rely on ASNS in order to produce asparagine. ECs synthesize asparagine by converting glutamine-derived nitrogen and aspartate [114,115,116,117,118]. Therefore, GLS1 and ASNS are interesting metabolic targets to be considered in the antiangiogenic strategies.

Treatment with the FASN blocker orlistat caused antiangiogenic effects already at lower doses than that needed to impair cancer cell growth, offering a therapeutic window to use FASN inhibitors as an alternative antiangiogenic agent in cancer patients [12]. In agreement, in mice lacking endothelial FASN (FASN^ΔvEC^), angiogenesis was impaired, and treatment with a pharmacological FASN inhibitor reduced pathological ocular neovascularization by decreasing EC proliferation [12]. Moreover, FASN inhibitors have antilymphangiogenic effects, decreasing LEC viability, proliferation, and migration, thereby impairing cancer cell lymphatic metastasis [119].

ECs use serine for the generation of dTTP, dATP and dGTP. PHGDH inhibition also impairs dCTP production indirectly as a consequence of reduced mitochondrial respiration, and impairs glycine production as a result of serine depletion [14]. Glycine is then required for heme synthesis in ECs, and the latter is crucial for the proper functioning of several enzymes, including the complexes II, III, and IV of OXPHOS mitochondrial respiration. As a result, the activity of mitochondrial dihydroorotate dehydrogenase, an enzyme involved in a key step in the production of dCTP, is decreased. Endothelial PHGDH silencing triggered severe oxidative stress, mitochondriopathy and impaired EC proliferation and sprouting in vitro. Moreover, decreased angiogenesis and neonatal lethality was observed in PHGDH^ECKO^ pups [14].

Genetic loss of OXCT1 in LECs impairs lymphangiogenesis, while ketone-body supplementation induces lymphatic growth in both developmental and pathological conditions [83]. In a mouse model of surgical ablation of lymphatic vessels in the tail, recapitulating characteristics of acquired lymphedema in humans, the ketogenic diet improved lymphatic vessel growth and lymph drainage and reduced lymphedema, suggesting a novel therapeutic opportunity through dietary metabolite supplements [1,83]. These findings may warrant future exploration of OXCT1 for inhibition of tumor-associated lymphangiogenesis and prevention of lymphatic metastasis.

## 6. Concluding Remarks and Future Perspectives

Mounting evidence supports the idea that targeting both the “seed” (cancer cells) and the “soil” (TEM) can be the most advantageous approach for cancer therapy. In this context, the development of more effective antiangiogenic strategies and their combination with other antitumor drugs are receiving great attention. Despite relevant research progress, the complex interactions between cancer cells and stromal cells and the metabolic reprogramming occurring in the TEM components are not fully understood. Recent studies have provided insight into the EC metabolism and have unveiled the therapeutic potential of druggable metabolic pathways and enzymes in angiogenesis (Figure 4a). Moreover, the use of multi-omics technologies has illustrated the metabolic heterogeneity of individual ECs in health and disease, or in different tissue types. Considering the unique EC metabolic features and the benefits of tumor vasculature normalization, multiple opportunities are available to develop alternative antiangiogenic therapies (Figure 4b,c). Not only approaches targeting TEC metabolism as monotherapy should be considered, but also, the use of combination strategies targeting “escape” EC metabolic pathways, such as chemo-, radio-, and/or immune therapy. The improved antiangiogenic therapies in combination with antitumor drugs offer multiple opportunities in clinical practice.

## Data Availability

Not applicable.
